# Post-Operative Urinary Tract Infections After Radical Cystectomy: Incidence, Pathogens, and Risk Factors

**DOI:** 10.3390/jcm13226796

**Published:** 2024-11-12

**Authors:** Maxwell Sandberg, Rachel Vancavage, Justin M. Refugia, Gavin Underwood, Emily Ye, Claudia Marie-Costa, Rainer Rodriguez, Nicos Prokopiou, Randall Bissette, Ronald Davis III, Ashok Hemal, Alejandro R. Rodriguez

**Affiliations:** 1 Department of Urology, Wake Forest University School of Medicine, Winston Salem, NC 27101, USAalrrodri@wakehealth.edu (A.R.R.); 2 Albany Medical College, Albany, NY 12208, USA; vancavr@amc.edu (R.V.);; 3 Wake Forest University School of Medicine, Winston Salem, NC 27101, USA; gwunderw@wakehealth.edu (G.U.);; 4 Virginia Tech Carilion School of Medicine, Roanoke, VA 24016, USA; rgbissette@gmail.com

**Keywords:** radical cystectomy, bladder cancer, urinary tract infection, bacteria, antibiotics

## Abstract

**Background**: The incidence of urinary tract infections (UTIs) after radical cystectomy (RC) with urinary diversion (UD), the typical pathogens, and associated patient risk factors have not been well documented. In this study, we examined the incidence of post-op UTIs after RC to identify associated risk factors. **Methods**: Single-center, retrospective case series of 386 patients with bladder cancer who underwent RC with UD between 2012 and 2024. The primary objective was UTI incidence, defined by the frequency of patients with urine culture with >10^5^ colony-forming units per high-powered field, spanning from post-op day 0 (POD0) to 90 days after discharge. Isolated pathogens were reported. Risk factors for UTIs were assessed. **Results**: The average age was 69 years old at surgery, and patients were predominantly male (80%). The cumulative incidence of post-op UTIs was 14%, among which 12 patients had more than one UTI. The UTI incidence was 2%, 8%, and 7% during the immediate post-op period, within 30 days, and within 31–90 days, respectively. Isolated pathogens included *Escherichia coli* (26%), *Enterococcus faecalis* (24%), *Klebsiella pneumoniae* (21%), and *Pseudomonas species* (21%). In the immediate post-op period, female sex was the only significant risk factor. At 31 to 90 days, cutaneous ureterostomy UD was the predominant risk factor for UTIs. For ileal conduit patients, those with a Wallace ureteral anastomosis were associated with UTI 31–90 days from discharge for RC. **Conclusions**: Our retrospective data suggests the incidence of UTIs and their causative pathogens after RC differ based on post-operative time points and vary according to different patient risk factors.

## 1. Introduction

Radical cystectomy (RC) for bladder cancer carries a high morbidity and mortality rate postoperatively. Complication rates vary but are estimated at ~34.9% in-house, ~39% within 30 days after RC, and ~58.5% within 90 days of RC [1]. Shabsigh et al. attempted to classify RC complications into eleven different categories in what has become known as the Memorial Sloan-Kettering Cancer Center (MSKCC) complication grading system [2]. Aside from gastrointestinal complications, infectious etiologies are the most likely to be seen within 90 days of RC using the MSKCC criteria, and the overwhelming majority of these tend to be urinary tract infections (UTIs) [2].

UTIs after RC lead to many additional complications for both patients and the healthcare system. This includes emergency department utilization, readmission, multi-organ dysfunction, urosepsis, and even death. The rate of incidence varies in the literature, ranging from ~11–36% [3,4,5]. Despite being relatively common and causing many issues for patients and providers, there is a lack of high-powered research into UTIs after RC. Prior studies seem to indicate that either *Escherichia (E.) coli* or *Enterococcus faecalis* is the most common pathogen isolated in culture, but this remains uncertain [6,7,8]. The use of antibiotic prophylaxis in the perioperative period and its effect on UTI also remains controversial [9,10]. Researchers have focused on developing models to predict UTI occurrence after RC. Some evidence points towards continent diversions, a history of diabetes mellitus (DM), and a greater comorbidity index as risk factors [8,11]. Further exploration of a comprehensive set of risk factors is warranted. The purpose of this study was to identify the rate of UTI after RC for bladder cancer in a large cohort of patients and identify the most common pathogens seen in urinary culture. The secondary purpose of this study was to identify risk factors for UTI after RC and analyze how UTI affects patient survival for those afflicted with bladder cancer.

## 2. Materials and Methods

A single-center retrospective analysis of all patients with a diagnosis of bladder cancer, specifically urothelial carcinoma, who underwent RC between 2012 and 2024 was conducted. All patients who underwent RC for a reason other than bladder cancer were excluded. Clinicopathologic data points included age at RC, sex, race/ethnicity, medical comorbidities (hypertension and diabetes mellitus), and the Charlson Comorbidity Index (CCI). Operative and peri-operative data points were operative approach, operative time, urinary diversion type, hospital length of stay, postoperative complications (graded according to the Clavien-Dindo classification system), and post-operative follow-up. Urinary diversion was defined by the presence of an ileal conduit, neobladder, cutaneous ureterostomy, or percutaneous nephrostomy tube(s) (PCN). For those with an ileal conduit, the ureteral anastomosis method was also recorded as Bricker or Wallace Type 1 (no patients received Wallace Type 2). All patients had a preoperative urine culture to rule out the presence of an active UTI at the time of surgery. UTIs were tracked in the immediate postoperative period prior to discharge, within 30 days after discharge, and 31–90 days after discharge. Patients required a urine culture with >10^5^ colony forming units/high-powered field to be deemed UTI positive or unavailable/negative urine culture, but the clinical presumption of UTI, including fever, flank pain, or leukocytosis, in a definition like that of Ghoreifi et al. [3]. Febrile UTI was a temperature ≥ 101.5° Fahrenheit in the immediate postoperative period and ≥100.5° Fahrenheit after discharge from the hospital. Urosepsis was defined according to the Third International Consensus Definitions for Sepsis and Septic Shock (Sepsis-3) [12]. All bacteria and/or fungi causing each UTI were also recorded. The category of “other” represented any pathogens that were isolated on only one urine culture, and any pathogens isolated on two or more cultures were categorized separately. All patients received perioperative antibiotic prophylaxis at the time of RC. Additional perioperative antibiotics were at the operating surgeon’s discretion prior to the year 2018. After 2018, an enhanced recovery after surgery protocol (ERAS) was introduced, and all patients received antibiotics after surgery, either cefazolin, ceftriaxone, or cefoxitin for three days. In addition, patients received postoperative bowel stimulation with alvimopan until the return of bowel function was documented, ureteral stents, and venous thrombosis prophylaxis during admission and out to 28 days from surgery at discharge. Ureteral stents in urinary diversions were removed at the surgeon’s discretion. To be documented with a ureteral stricture in a urinary diversion after RC, an imaging study was required for confirmation, most often a computerized tomography scan. Vesicoureteral reflux (VUR) diagnosis was based on a combination of imaging study and documentation in the patient’s medical record. Bowel resection at the time of RC most often occurred due to either bowel injury intraoperatively or concomitant colorectal cancer.

To analyze risk factors for UTIs, patients with and without a UTI were compared using a variety of patient variables at each time interval in the study. Ureteral stent placement and removal times were not accurately documented in the medical record prior to the year 2018, so this was not included as a risk factor in the analysis. Statistical analysis was conducted using independent samples t-test, chi-squared test, and Kaplan-Meier survival analysis with significance set to *p* < 0.05 using SPSS Statistics Version 28 (Armonk, NY, USA). Specifically for survival analysis, overall survival was evaluated between patients who did and did not develop a UTI within 30 days of RC and 31–90 days after RC. A binary logistic regression model was also run with UTI as the outcome any time after RC with significance set to *p* < 0.05. Variables seen on univariable analysis with *p* < 0.05 and/or established clinical relevance were included in the model to produce an optimized model that fit the data without collinearity.

## 3. Results

There were 386 patients included in the study. Of these, 308 were male and 78 females. The overall incidence of UTI was 13.7% (N = 53). Seven (1.8%) patients developed a UTI during the immediate postoperative period from RC, 30 (7.8%) patients had a UTI within 30 days of discharge from RC and 25 (6.5%) patients within 31–90 days of discharge from RC. Twelve (3.1%) patients had a recurrent UTI at multiple time points in the study. Urosepsis was present in eight (15.1%) patients. Fifty-four (87%) patients had at least one symptom associated with their presentation, based on our clinical definition of UTI. Of all patients with a documented UTI, two (3.8%) had a documented history of VUR, and eight (15.1%) had a history of ureteral stricture after RC in their urinary diversion.

Six of the seven (85.7%) patients with a UTI in the immediate postoperative period received antibiotic prophylaxis while admitted for recovery during their hospital stay (Table 1). Antibiotics were not standardized in the cohort, and the most common ones were cefoxitin (N = 3), cefazolin (N = 2), and ceftriaxone (N = 1). The mean time of antibiotic duration before UTI was 4.5 days from RC. The mean age at surgery was 70.4 years for patients with a UTI in the immediate postoperative period, which was not significantly different from those without a UTI, whose mean age was 65.2 (*p* = 0.381). Four of the seven (57.1%) patients were female in the UTI group compared to 74 (19.5%) patients who did not develop an immediate postoperative UTI, which was a significantly greater proportion (*p* = 0.014). No significant differences in race, CCI, or comorbidities were seen between those with and without a UTI (*p* > 0.05). Operative approach (*p* = 0.454), concomitant nephroureterectomy (*p* = 0.654), and diversion type (*p* = 0.182) were similar between patients with and without immediate postoperative UTI. For those with an ileal conduit, UTI was not different between the Bricker and Wallace anastomosis (*p* = 0.314). Bowel resection at the time of RC was associated with UTI in the immediate postoperative period (*p* = 0.034). Five of the 7 patients (71.4%) with a UTI in the immediate postoperative period were febrile. Urosepsis was seen in one patient (14.3%). The most frequently seen bugs in urine culture were *Klebsiella pneumoniae* (N = 4), “other” pathogens (N = 4), *E. coli* (N = 3), and Pseudomonas (N = 3). Tumor pathology was similar amongst patients with and without a UTI as well (*p* = 0.413). Incidence of death (*p* = 0.494), overall survival (*p* = 0.453), incidence of cancer-specific death (*p* = 0.324), and cancer-specific survival (*p* = 0.169) also did not differ between those with and without a UTI in the immediate postoperative period from RC.

**Figure 1:**
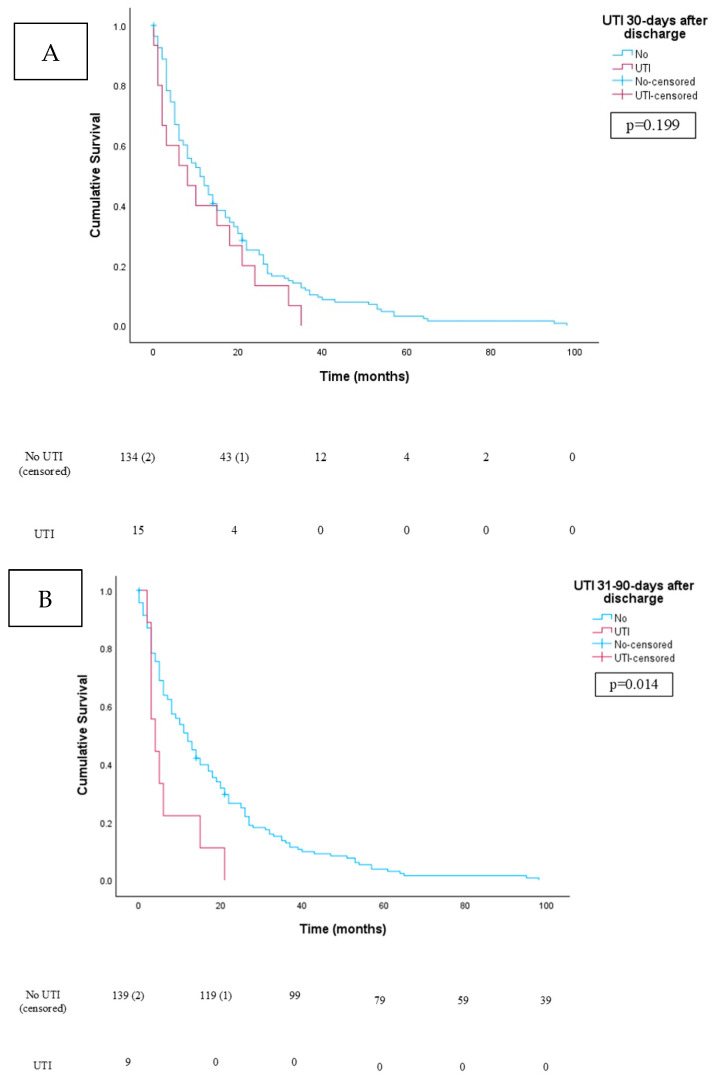
Kaplan-Meier survival curve. The following table represents a comparison of overall survival between patients who did and did not develop a UTI at both 30– (**A**) and 31–90 days (**B**) after discharge from radical cystectomy. Patients who formed a UTI are on the red line, and those who did not form a UTI are on the blue line. The number at risk table is also provided below with time in months. There was a statistically significant difference in overall survival, favoring patients who did not develop a UTI 31–90 days after discharge using the log-rank test (*p* = 0.014).

**Figure 2:**
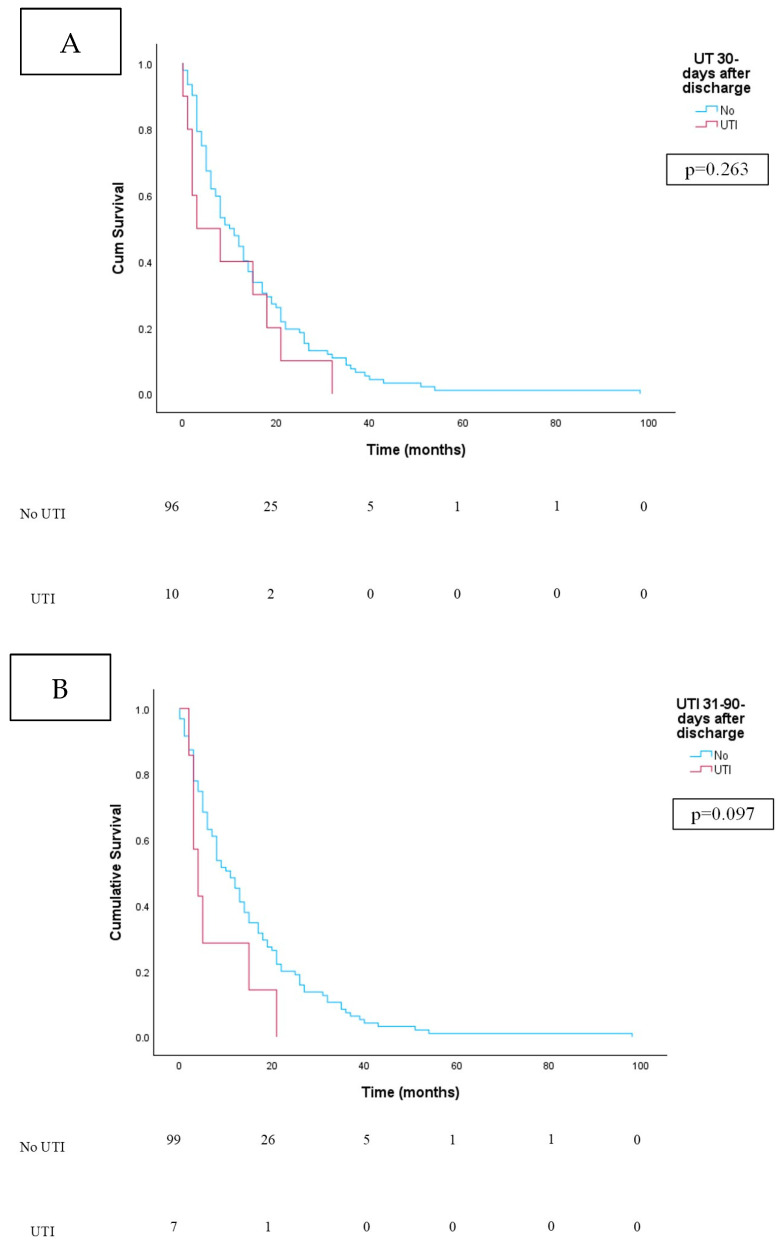
Kaplan-Meier survival curve. The following table represents a comparison of cancer-specific survival between patients who did and did not develop a UTI at both 30- (**A**) and 31–90 days (**B**) after discharge from radical cystectomy. Patients who formed a UTI are on the red line, and those who did not form a UTI are on the blue line. The number at risk table is also provided below with time in months. There was not a statistically significant difference in cancer-specific survival (*p* > 0.05).

For patients with a UTI within 30 days of discharge from the hospital, the mean age at surgery was 65.2 years and was like those without a UTI in this period, whose mean age was 66.9 years (*p* = 0.475) (Table 1). There was a significant difference in race, with “other” races being more likely in the non-UTI group (*p* = 0.013). CCI and prevalence of diabetes were similar in the UTI and non-UTI cohorts 30 days after discharge (*p* > 0.05). Hypertension was more prevalent in those who developed a UTI 30 days from discharge (*p* < 0.001). Approach (*p* = 0.352), concomitant nephroureterectomy (*p* = 0.219), and diversion type (*p* = 0.977) did not differ significantly in those with and without a UTI 30 days from discharge. For those with an ileal conduit, UTI was not different between the Bricker and Wallace anastomosis (*p* = 0.643). Twenty-two (73%) patients with a UTI 30 days from discharge were febrile at presentation, and the most common pathogens seen in urine culture were *Enterobacter cloacae* (N = 8), *E. coli* (N = 6), and *Pseudomonas* (N = 5). Urosepsis was present in four (13.3%) patients. Tumor pathology (*p* = 0.335), incidence of death (*p* = 0.372), overall survival (*p* = 0.320), incidence of cancer-specific death (*p* = 0.915), and cancer-specific survival (*p* = 0.343) were also similar. On survival analysis, no difference was seen in overall survival time between those with and without a UTI 30 days from discharge (Figure 1A; *p* = 0.199). Similarly, on survival analysis, cancer-specific survival was not different in those patients who did and did not develop a UTI 30 days from discharge (Figure 2A; *p* = 0.263).

Mean age at surgery was similar between patients with (66.9 years) and without (66.5 years) a UTI 31–90 days after RC discharge (*p* = 0.891) (Table 1). No significant differences in gender, race, CCI, or diabetes were seen between those with and without a UTI (*p* > 0.05). Hypertension was significantly more prevalent in the stone-forming group (*p* = 0.030). Approach (*p* = 0.082) and concomitant nephroureterectomy (*p* = 0.386) did not differ significantly in those with and without a UTI 31–90 days from discharge. Bowel resection during RC was more likely to be performed in patients who developed a UTI 31–90 days after RC (*p* = 0.030). Twenty-one (84%) patients were febrile at presentation. Urosepsis was seen in three (12%) patients. The most common pathogens in urine culture were *E. coli* (N = 5) and *Klebsiella pneumoniae* (N = 5), followed by Pseudomonas (N = 3), *Enterobacter cloacae* (N = 3), and *Enterococcus faecalis* (N = 3). Patients who developed a UTI 31–90 days after RC were more likely to have a cutaneous ureterostomy diversion (*p* = 0.001). For those with an ileal conduit, UTI was more common in those with a Wallace than Bricker ureteral anastomosis (*p* = 0.033). Tumor pathology (*p* = 0.162), incidence of death (*p* = 0.511), incidence of cancer-specific death (*p* = 0.881), and cancer-specific survival (*p* = 0.203) were similar. Overall survival was worse in patients who developed a UTI in this time frame (*p* = 0.001). On survival analysis, overall survival was worse in those patients who developed a UTI 31–90 days from discharge (Figure 1B; *p* = 0.014). On survival analysis, cancer-specific survival was not different in those patients who did and did not develop a UTI 31–90 days from discharge (Figure 2B; *p* = 0.097).

On multivariable logistic regression, diversion type, gender, and bowel resection during RC were not significant risk factors for UTI (Table 2). Hypertension was significantly associated with 4.1 times increased odds of UTI any time after RC (*p* < 0.001).

## 4. Discussion

UTI after RC is a common complication with significant morbidity for patients. This is not surprising, given that RC carries a significant complication rate, estimated as high as 60%, and of this, around 15% are Clavien grade III-V [13,14]. Prior studies have put UTI incidence within 90 days of RC at 11–36% [3,5,8,9]. UTI prevalence was 13.7% in our study population, with the majority of UTIs occurring in the 30-day window after discharge from the hospital. Most patients also presented with a fever when they were diagnosed with their UTI in our study. Urosepsis rates were also within the range of other publications, showing that most patients who develop a UTI after RC do not present with/go on to develop urosepsis [3,8]. Ureteral stricture rates were higher in our cohort than most publications, but despite being a known risk factor for UTI in RC patients, it does not appear UTIs occurred at a greater rate in our population than that reported in the literature [4,8]. The rate of VUR was low in RC patients with a UTI, as only two had it documented in their medical records. Other studies have examined videourodynamics in RC patients after surgery, finding higher rates of VUR as great as 42% and linking this to a risk factor for UTI [4]. Most patients did not have videourodynamics in our cohort, so we were unable to assess this, which is a limitation of our analysis, potentially underestimating the true rate of VUR in our cohort. The significant advantage, though, of our study is the analysis of UTI at three separate time points: immediately after RC, within 30 days of discharge, and 31–90 days from discharge.

UTIs were uncommon in the immediate postoperative period, and the low event rate makes comprehensive analysis difficult. Nevertheless, several observations are worth discussing in the study cohort. All patients had a dose of antibiotics at the time of surgery, and perioperative antibiotic prophylaxis was still used in most patients with a UTI after RC and after our ERAS protocol initiation in 2018 in all patients. The utility of antibiotic prophylaxis after RC is highly debated. Hara et al. compared one versus three days of prophylactic antibiotics after RC, showing no difference in febrile UTI rates [15]. The mean time of antibiotic prophylaxis in our study before UTI was 4 days. Antonelli et al. performed a systemic metanalysis of preoperative antibiotic prophylaxis for RC, and no definitive benefit was found in infection prevention or specific regimens [16]. Interestingly, Werntz et al. studied postoperative antibiotic prophylaxis after discharge from RC and found it significantly decreased rates of UTI, urosepsis, and hospital readmission [17]. More research is needed on this topic in an effort towards antibiotic stewardship. The most common pathogens seen in culture were “other” pathogens and *Klebsiella pneumoniae,* followed by *E. coli/Pseudomonas*. Kim et al. noted that *E. coli* was the most common bug isolated in urine culture in their study of UTI after RC but did not analyze UTIs during the initial hospital stay [4]. Lu et al. found that 12% of their RC cohort with UTI were caused by *Klebsiella pneumoniae* but similarly lacked information in the immediate postoperative period that we include [8]. Haider et al. published that *Klebsiella pneumoniae* was the second most common cause of UTI after RC behind Enterococcus species, and Antonelli et al. similarly found Enterococcus species to be the primary cause of UTI in their metanalysis [9,16]. Our results indicate that the pathogenic cause of UTI after RC may depend on when a patient presents after surgery, challenging the dogma that *E. coli* and Enterococcus species are always the primary culprits. Lastly, it is important to address the gender discrepancy we found in UTIs immediately after surgery, favoring females. Wood et al. have also noted that female gender may be a risk factor for UTI after RC, but other studies have found no such association [8,11,18]. As we only had seven UTIs prior to discharge, it is difficult to say whether gender is truly linked to immediate postoperative UTI, but given that gender has been linked to UTI in the general population, it also calls for further investigation.

UTIs were most seen in the 30 days after discharge from RC. Ghorefi et al. found the median time for UTI identification was 13 days after RC [3]. Aldhaam et al. similarly found that 48% of all UTIs in their robotic RC cohort occurred within 30 days of surgery [6]. It appears that patients are most at risk for UTI after initial discharge from the hospital for RC. The most frequent bugs isolated in urine culture were *Enterococcus faecalis*, *E. coli*, and *Pseudomonas*, which appears like other publications’ conclusions [3,4,5,6,8,9]. Two patient variables were also associated with UTI within 30 days of discharge, namely hypertension and bowel resection at the time of RC. Treatment with antihypertensive medications and a diagnosis of hypertension has been studied to assess links to UTIs, with mixed results [19,20]. However, we ultimately feel we cannot conclude that hypertension is a risk factor for UTI due to the heterogeneous study population we have where multiple established risk factors for perioperative UTI were not standardized, such as stent management and perioperative antibiotic use. Bowel resection at the time of RC was also associated with UTI 30 days from discharge, along with in the immediate postoperative period and 31–90 days from RC. This is a novel risk factor we identified in our study population, but the link between bowel resection and UTI is well-established in patients undergoing colorectal surgery for cancer [21,22]. Nevertheless, bowel resection did not remain significant on multivariable logistic regression, which limits the strengths of our conclusions here.

The most common pathogens causing UTI 31–90 days from discharge for RC were *E. coli* and Klebsiella, followed by *Pseudomonas*, *Enterobacter cloacae*, *Enterococcus faecalis*, and “other” species. As noted previously, this seems to be in line with the expected results from the literature. One of the most important findings was the link between urinary diversion and UTI in this time frame, specifically cutaneous ureterostomy. Orthotopic neobladder has been shown to be a risk factor for UTI after RC in multiple studies [3,5,7]. The authors hypothesize that excessive mucus production from the bowels used for neobladders leads to increased risk [23]. Although less known, cutaneous ureterostomy has also been linked to UTI after RC, thought to be due to the higher stricture rate in this urinary diversion [24,25]. Unsurprisingly, in our cohort, 50% of all strictures in patients with a UTI also had a cutaneous ureterostomy diversion. Interestingly, when comparing Bricker and Wallace ureteral anastomoses for ileal conduit patients, the rate of UTI 31–90 days from RC was greater in those with a Wallace anastomosis. Few have looked at UTI rates in ileal conduits by anastomosis type, but strictures, a risk factor for UTI after RC, are studied in the literature. Some have noted the stricture rate to be worse with the Wallace technique, but a recent meta-analysis revealed no difference in stricture rates between the two [26,27]. Our finding calls for further investigation into UTI between the two techniques, where there is an apparent gap in the literature currently. Overall survival after RC was also significantly lower in patients with a UTI 31–90 days from RC. While the UTI itself is unlikely to be the sole reason for worse survival in these patients, it may serve as a warning sign of a complicated postoperative course and the need for more diligent postoperative monitoring. This is also reinforced by the fact that cancer-specific survival was not different in this cohort. It is also important to recognize that the inflammatory response of the body to UTI could also play a role in impacting overall survival, and Volz et al. have shown that perioperative inflammatory markers correlate with survival outcomes after RC [28].

This study has several limitations that are important to acknowledge. First, it is subject to the same inherent biases any retrospective review has, especially selection bias in the study cohort. It is certainly possible more UTIs were present in the cohort, and these patients either never had a urine culture and/or presented to an institution outside of our own, which we did not capture. Preoperative markers of inflammation would also have been useful to assess and include in our regression model, but these were not readily available in the medical record. Additionally, we did not have a complete set of sensitivities from patient urine cultures, so this was not included, which would have strengthened the results. Moreover, the low event rate for UTI in the immediate postoperative period makes definitive analysis difficult. Further, it is well established that patients with urinary diversions have urinary colonization at baseline, making it difficult to distinguish between a real UTI versus colonization in the study population. However, the majority of patients in the study had at least one symptom as employed in our definition of UTI plus or minus a positive urine culture, making it far more likely these were real UTIs. We feel the decision to separate UTIs into three-time points is a strength of this study, but others may disagree with this decision or the time cutoffs we elected to use. We attempted to identify a comprehensive list of potential risk factors for UTI but did not include ureteral stent placement/removal as there was inconsistent documentation in the medical record prior to the year 2018, which also limits our findings. Some have noted that stent placement is a risk for UTI after RC, as is the extended duration of its use postoperatively [29,30]. However, Veccia et al. recently conducted a meta-analysis comparing stented RC patients to stent-free RC patients, noting no significant difference in the rate of postoperative UTI or stricture [31]. Prospective studies are needed to confirm the results discussed in this manuscript.

## 5. Conclusions

UTI is a perplexing problem many patients are likely to face after RC, yet there are no proper guidelines on how to try and prevent, manage, and identify patients most at risk for them. The utility of this study lies in its ability to analyze UTIs at three separate time points postoperatively from RC, showing that pathogens and risk factors differ based on time of presentation. In the immediate postoperative period, female gender may predispose patients to a UTI, and whether prophylactic antibiotics are useful after surgery remains unclear. The 30-day window from RC is when patients are most likely to have a UTI, and both hypertension and bowel resection are associated with UTI. These same findings appear to hold true for UTI 31–90 days from RC. On multivariable analysis, though, only hypertension remained significant, and we feel it cannot be concluded that this is a true risk factor for UTI. Additionally, cutaneous ureterostomy was identified as a risk factor in this time frame. The ureteral anastomosis technique also needs more study based on our analysis. Moreover, overall survival appears to be related to the development of a UTI, which may serve as an early warning sign for a complicated postoperative course. UTI remains a vexing problem for patients, practitioners, and the healthcare system alike after RC, which calls for more large-scale studies to be properly managed.

## Figures and Tables

**Table 1:** Risk factors for urinary tract infection. The following table shows the demographic characteristics of patients who formed a UTI at each time point in the study. Deaths, any cause, represent the total number of patients who died in each cohort by the end of the study window. Cancer-specific death is represented in a similar manner. Associated *p*-values are also provided to compare potential risk factors for each cohort between patients with and without a UTI.

Variable	UTI Immediately Post-Op			UTI within 30 d of Discharge			Uti within 31–90 d of Discharge		
	Yes	No	*p*-Value	Yes	No	*p*-Value	Yes	No	*p*-Value
N	7	379	-	30	356	-	25	361	-
Culture confirmed	6	-	-	25	-	-	23	-	-
Fever	5	-	-	22	-	-	21	-	-
Urosepsis	2	-	-	5	-	-	1	-	-
Uropathogen									
*E. coli*	3	-	-	6	-	-	5	-	-
*K. pneumoniae*	4	-	-	2	-	-	5	-	-
*Pseudomonas* spp.	3	-	-	5	-	-	3	-	-
*S. marcesenes*	1	-	-	0	-	-	1	-	-
*S. epidermidis*	2	-	-	4	-	-	2	-	-
*S. aureus*	0	-	-	3	-	-	2	-	-
*E. clocae*	0	-	-	2	-	-	3	-	-
*E. faecalis*	2	-	-	8	-	-	3	-	-
*K. oxytocae*	2	-	-	2	-	-	0	-	-
*Proteus* spp.	0	-	-	1	-	-	0	-	-
Other	4	-	-	3	-	-	3	-	-
Female sex	4	74	0.014	6	62	0.883	4	63	0.708
Age at surgery, yrs	70.4	66.7	0.318	65.2	66.9	0.475	66.9	66.5	0.891
Race			>0.99			0.013			0.561
Caucasian	6	331		24	286		21		
African American	1	37		4	33		3		
Hispanic	0	3		1	2		1		
Other	0	8		1	7		0		
Charlson Comorbidity Index	5.9	5.3	0.448	4.9	5.2	0.403	5.3	5.1	0.635
History of hypertension	5	198	0.314	25	158	<0.001	18	168	0.030
History of diabetes	2	90	0.767	9	77	0.423	8	78	0.342
Surgical approach			0.454			0.352			0.082
Open	3	216		20	190		19	193	
Robotic	4	163		10	138		6	138	
Concomitant procedures									
Nephroureterectomy	0	17	0.654	0	17	0.219	0	15	0.386
Bowel resection	1	9	0.034	3	6	0.013	2	7	0.030
Urinary diversion			0.182			0.977			0.001
Ileal conduit	6	311		24	269		14	276	
Orthotopic neobladder	1	7		1	7		0	8	
Cutaneous ureterostomy	0	50		43	4		10	37	
Percutaneous nephrostomy tube(s)	0	9		1	8		1	8	
Ileal conduit ureteral anastomosis Bricker Wallace	6 0	266 45	0.314	17 4	228 41	0.643	12 6	231 39	0.033
Cystectomy pathology			0.413			0.335			0.162
T0	0	45		3	41		1	43	
Ta	1	12		0	12		0	12	
Tis	0	28		2	25		0	27	
T1	1	29		0	26		1	24	
T2a	0	63		7	50		33	54	
T2b	2	53		6	48		3	51	
T3a	2	48		5	43		4	43	
T3b	1	31		4	24		5	23	
T4a	0	59		1	51		6	46	
T4b	0	8		1	6		1	6	
Deaths, any cause	40	167	0.494	15	136	0.372	9	141	0.511
Overall survival, mos	13.3	17.4	0.453	11.9	16.6	0.320	6.9	17.2	0.001
Cancer-specific death	2	113	0.324	10	96	0.915	7	99	0.881
Cancer-specific survival, mos.	14.5	0.50	0.169	10.2	14.9	0.343	7.6	14.9	0.203

**Table 2:** Multivariable logistic regression. The following table is a regression model with the outcome being urinary tract infection at any time after radical cystectomy. For conduit, the ileal conduit is the referent group. The constant in the model represents the predicted value of the dependent variable (UTI at any time) when all other independent variables are equal to zero.

Variable	B	Standard Error	df	Significance	Exp(B)	95% CI	
						Lower	Upper
Bowel Resection	1.2	0.7	1	0.077	3.3	0.9	12.7
Conduit	-	-	4	0.802	-	-	-
Ileal conduit (referent)	-	-	-	-	-	-	-
Neobladder	1.6	0.9	1	0.069	5.0	0.9	28.5
Cutaneous ureterostomy	0.5	0.4	1	0.189	1.7	0.8	3.8
Percutaneous nephrostomy	0.08	1.1	1	0.946	1.1	0.1	9.3
Female sex	0.3	0.4	1	0.446	1.3	0.6	2.7
Hypertension	1.4	0.4	1	<0.001	4.1	2.0	8.7
Constant	−3.0	0.4	1	<0.001	0.05	-	-

## Data Availability

Data is available upon request to the corresponding author. It is not publicly available due to confidentiality reasons.
